# Ultra-fast whole-brain T2-weighted imaging in 7 seconds using dual-type deep learning reconstruction with single-shot acquisition: clinical feasibility and comparison with conventional methods

**DOI:** 10.1007/s11604-025-01875-6

**Published:** 2025-09-26

**Authors:** Yohei Ikebe, Noriyuki Fujima, Hiroyuki Kameda, Taisuke Harada, Yukie Shimizu, Jihun Kwon, Masami Yoneyama, Kohsuke Kudo

**Affiliations:** 1 Department of Diagnostic and Interventional Radiology, Hokkaido University Hospital, Kita-Ku, Sapporo, 0608638 Japan; 2 Center for Cause of Death Investigation, Faculty of Medicine and Graduate School of Medicine, Hokkaido University, Sapporo, Japan; 3 Department of Diagnostic Imaging, Faculty of Medicine and Graduate School of Medicine, Hokkaido University, Sapporo, Japan; 4 Faculty of Dental Medicine, Department of Radiology, Hokkaido University, Sapporo, Japan; 5 Department of Advanced Diagnostic Imaging Development, Faculty of Medicine and Graduate School of Medicine, Hokkaido University, Sapporo, Japan; 6 Philips Japan, Tokyo, Japan

**Keywords:** Brain, MRI, Deep learning reconstruction, Super-resolution, Single-shot turbo spin-echo

## Abstract

**Purpose:**

To evaluate the image quality and clinical utility of ultra-fast T2-weighted imaging (UF-T2WI), which acquires all slice data in 7 s using a single-shot turbo spin-echo technique combined with dual-type deep learning (DL) reconstruction, incorporating DL-based image denoising and super-resolution processing, by comparing UF-T2WI with conventional T2WI.

**Material and methods:**

We analyzed data from 38 patients who underwent both conventional T2WI and UF-T2WI with the dual-type DL-based image reconstruction. Two board-certified radiologists independently performed blinded qualitative assessments of the patients’ images obtained with UF-T2WI with DL and conventional T2WI, evaluating the overall image quality, anatomical structure visibility, and levels of noise and artifacts. In cases that included central nervous system diseases, the lesions’ delineation was also assessed. The quantitative analysis included measurements of signal-to-noise ratios in white and gray matter and the contrast-to-noise ratio between gray and white matter.

**Results:**

Compared to conventional T2WI, UF-T2WI with DL received significantly higher ratings for overall image quality and lower noise and artifact levels (*p* < 0.001 for both readers). The anatomical visibility was significantly better in UF-T2WI for one reader, with no significant difference for the other reader. The lesion visibility in UF-T2WI was comparable to that in conventional T2WI. Quantitatively, the SNRs and CNRs were all significantly higher in UF-T2WI than conventional T2WI (*p* < 0.001).

**Conclusion:**

The combination of SSTSE with dual-type DL reconstruction allows for the acquisition of clinically acceptable T2WI images in just 7 s. This technique shows strong potential to reduce MRI scan times and improve clinical workflow efficiency.

## Introduction

Magnetic resonance imaging (MRI) is an important modality in neuroimaging, due to its superior soft-tissue contrast and noninvasive nature. Among the various sequences that are used with MRI, turbo spin-echo (TSE) T2-weighted imaging (T2WI) is an essential sequence in brain MRI protocols as it provides critical contrast for the assessment of pathological changes in neurological conditions. However, a persistent challenge of TSE imaging is its extended scan time, which can lead to motion-related artifacts and patient discomfort. These are particularly problematic for patients with limited ability to remain still, such as children, elderly patients, and critically ill patients. In addition, prolonged imaging times slow down patient processing, leading to inefficiencies in clinical operations.

The single-shot turbo spin-echo (SSTSE) sequence is a fast MRI technique that enables rapid imaging by acquiring data within a single repetition time (TR) [1]. This method significantly reduces the scan time, making it particularly useful for motion-prone subjects. However, it has limitations, including a reduced signal-to-noise ratio (SNR) with image blurring, primarily due to T2 decay during data acquisition, which can affect the overall image quality and diagnostic utility. For the reduction of T2 decay, reducing the number of shots by decreasing the spatial resolution may be a useful approach, but reducing the spatial resolution would lead to further greater image blurring.

Deep learning (DL) reconstruction has been introduced as an effective reconstruction technique; it uses deep neural networks to obtain superb image quality even in under-sampled data [2, 3]. A DL-based image reconstruction technique that incorporates additional DL super-resolution processing has recently emerged as a new type of DL-based reconstruction methodology. Several studies have demonstrated that the use of model-based DL processing in the image reconstruction cycle followed by a post-processing DL technique with a super-resolution function is an effective approach [4–12]. This dual-type DL reconstruction including a super-resolution function has the potential to improve image quality sufficiently even when the original data quality is poor. In particular, combining this technique with single-shot acquisition may allow high-quality imaging to be achieved while maintaining the fast scan time provided by single-shot methods.

We hypothesized that applying ultra-fast T2WI (UF-T2WI), which acquires image data from all slices using SSTSE techniques within a single repetition time (TR) of only 7 s, together with the dual-type DL-based image reconstruction method would allow for the acquisition of clinically sufficient images in a remarkably short time. We conducted the present study to evaluate the image quality of UF-T2WI with DL, compare its image quality with that afforded by conventional T2WI, and assess the new method’s feasibility and clinical utility.

## Material and methods

### Patients

The protocol of this retrospective study was approved by our institutional review board, and the requirement for patients’ written informed consent was waived. We selected eligible patients who had been examined at our hospital (Hokkaido University Hospital, Sapporo, Japan) during the months August through November 2024. based on the following inclusion criteria: (i) the patient was referred to our hospital and underwent brain MRI using a specific scanner (see below), (ii) MRI sequences including TSE-T2WIs (conventional T2WI and UF-T2WI) were performed, and (iii) image data of UF-T2WI reconstructed using the dual-type DL-based image reconstruction method were available. The results for 38 patients were included in our analyses. These patients who received both conventional T2WI and UF-T2WI were randomly assigned from among all patients undergoing brain MRI.

### Imaging parameters

All scanning was performed using a 3.0-Tesla MR unit (Ingenia Elition; Philips Healthcare, Best, Netherlands) with a 32-channel head coil. Two T2WI datasets were acquired: (i) 2D TSE-T2WI with conventional image reconstruction, and (ii) 2D UF-T2WI using the SSTSE technique. The acquisition parameters are summarized in Table 1. The acquisition time of UF-T2WI was markedly shortened accordingly (scan time: 1 min 20 s for the conventional T2WI, and 7 s for the UF-T2WI).

**Table 1:** The imaging parameters applied in the study

	Conventional	UF-T2WI with DL
Repetition time: TR (ms)	4000	7000
Echo time: TE (ms)	90	107.2
Field of view (mm)	240 × 198	240 × 196
Acquisition matrix	532 × 326	240 × 150
Reconstruction boxel (mm)	0.375 × 0.375	0.312 × 0.312
Reconstruction matrix	640 × 640	768 × 768
Slice thikness (mm)	5.0	5.0
Inter-slice gap (mm)	1.5	1.5
Flip angle (degrees)	90	90
Reduction factor	2.5	3.5
Number of slice	23	23
Scan time	1 min 20 s	7 s

TR: repetition time, TE: echo time, UF-T2WI with DL: ultra-fast T2WI with dual-type deep learning (DL)-based image reconstruction

### Data processing

We used a dual-type DL framework for UF-T2WI in which two convolutional neural networks (CNNs) operated independently during the image reconstruction process [11]. Initially, we applied a reconstruction model that integrated the DL architecture of the DL-based algorithm called Adaptive-CS-Net into the compressed-sensing (CS) sensitivity encoding (SENSE)-based iterative denoising cycle, which we refer to as the ‘model-based DL reconstruction’.

In this approach, Adaptive-CS-Net replaces the wavelet transform with a sparsifying transform in the CS denoising cycle. Effective image denoising was expected from this CNN-driven method with the Adaptive-CS-Net-based approach to sparsity with the image reconstruction of a CS denoising cycle. A detailed explanation of Adaptive-CS-Net and its denoising process is available [13].

The image data were then transferred to SuperRes-Net, the second network in this model. This model was trained using multiple pairs of originally high-resolution and secondary downscaled images with Gibbs ringing artifacts for the image resolution improvement and the removal of Gibbs ringing artifacts. The network-based process was performed in residual mode, which is common in super-resolution solutions; this mode repeatedly applies a sequence that includes a 2D convolutional layer followed by a rectifier layer [14]. The dual-type DL reconstruction method adopted for the present patients’ images incorporates technology from vendor prototypes (Philips NGSA patch), and all reconstruction processes were conducted within the MR console.

For the comparison, the conventional T2WI reconstruction that was also used consisted of SENSE, which is a well-established technique for parallel imaging, and post-processing with zero-filling interpolation (ZIP). The reduction factor for the conventional T2WI was 2.5, compared to 3.5 for the UF-T2WI.

### Image analysis: qualitative analyses (the reader study)

As a qualitative assessment, two board-certified radiologists with 19 and 11 years of experience in neuroradiology, respectively, visually evaluated the T2WI images (both conventional T2WI and UF-T2WI with DL) under blinded conditions, focusing on: (i) the overall image quality, (ii) the visibility of anatomical structures (including the visibility of the gray/white matter contrast), and (iii) the degrees of noise and artifacts. Each evaluation was conducted based on the following four-point Likert scale. 1 point: very poor, unavailable for diagnostic use. 2 points: poor, not unavailable but may affect diagnoses. 3 points: good, acceptable for diagnostic use. 4 points: excellent, almost no limitations for diagnostic use.

In cases in which central nervous system (CNS) diseases were observed, both T2WI sequences were referenced simultaneously, and UF-T2WI with DL was evaluated for its ability to delineate lesions compared to the conventional T2WI. Each evaluation was conducted based on the following four-point Likert scale. 1 point: very poor, the lesion is unclear and completely different from the conventional image. 2 points: poor, the lesion is partially different and unclear. 3 points: good, the lesion is nearly identical and acceptable. 4 points: excellent, equivalent to the conventional T2WI.

### Image analysis: quantitative analyses

Regions of interest (ROIs) for gray matter (GM) and white matter (WM) were defined to assess the SNRs for the WM and GM (SNRwm and SNRgm, respectively) and the contrast-to-noise ratios between GM and WM (CNRgm and CNRwm). The evaluation was performed on three cross-sections: at the level of the body of the lateral ventricle, at the basal ganglia, and at the inferior horn of the lateral ventricle. In each section, one ROI was placed in the gray matter and one in the white matter of each cerebral hemisphere, providing a total of 12 ROIs per patient. The approximate areas of all ROIs were < 8 mm^2^. Each ROI placed precisely using the DICOM viewer software EV Insite R (Public and Social Systems Solution Provider, Tokyo). The quantitative procedures were performed by a radiologist with 10 years of experience. SNRs and CNRs were calculated using the following formulae:

$$\begin{aligned} {\text{SNRwm }} = & {\text{ SI white matter}}/{\text{SD white matter}} \\ {\text{SNRgm }} = & {\text{ SI gray matter}}/{\text{SD gray matter}} \\ {\text{CNRgm}} - {\text{wm }} = & \, \left( {{\text{SI gray matter}}/{\text{SI white matter}}} \right)/{\text{SD white matter}} \\ \end{aligned}$$

where SI is the mean signal intensity, and SD is the standard deviation.

### Statistical analyses

The Shapiro–Wilk test was used for testing the normality of the data distribution for continuous variables. Qualitative scores were compared between two groups by the Wilcoxon signed-rank test. In the quantitative analysis, if the quantitative values followed a normal distribution, a paired t test was used to compare the two groups; if they did not, the Wilcoxon signed-rank test was applied. A *p* value < 0.05 was considered significant. All statistical analyses were performed using JMP Software (ver. 17.0.0; SAS Institute, Cary, NC, USA).

## Results

### Patient data

The characteristics of the 38 patients were as follows: 20 males and 18 females, with a median age of 61 years (range 9–88 years). Among the 38 cases, CNS diseases were observed in 24 cases. A summary of the patient characteristics is provided in Table 2.

**Table 2:** The characteristics of the study population (*n* = 38)

Total patients (n=38)	
Age	
Range	9-88
Median	61
Gender	
Male	20
Female	18
Presence of CNS diseases	
Present	24
Absent	14
Detail of CNS diseases	
Ischemic change	13
Old infarction	6
Glioma	2
Lymphoma	1
Metastasis	1
Edema due to AVM	1

CNS: central nervous system, AVM: arteriovenous malformation

### Qualitative analyses (reader study)

**Fig. 1:**
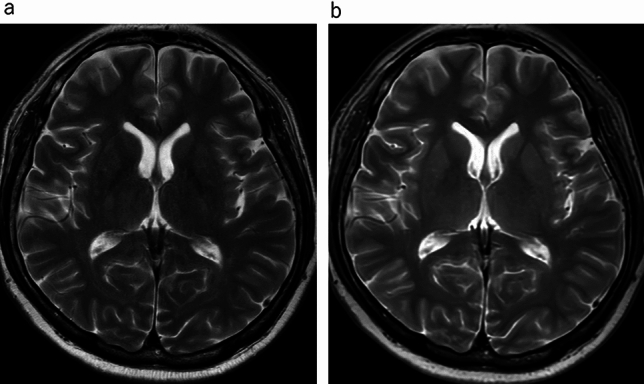
Representative case with normal imaging findings. Conventional T2-weighted image (T2WI) (**a**) and ultra-fast T2WI with dual-type deep learning (DL)-based image reconstruction (**b**) in the same patient. Overall, the visibility of major structures, including the cortex, white matter, basal ganglia, and thalamus, were more prominent in the DL-based ultra-fast T2WI compared to the conventional T2WI

**Fig. 2:**
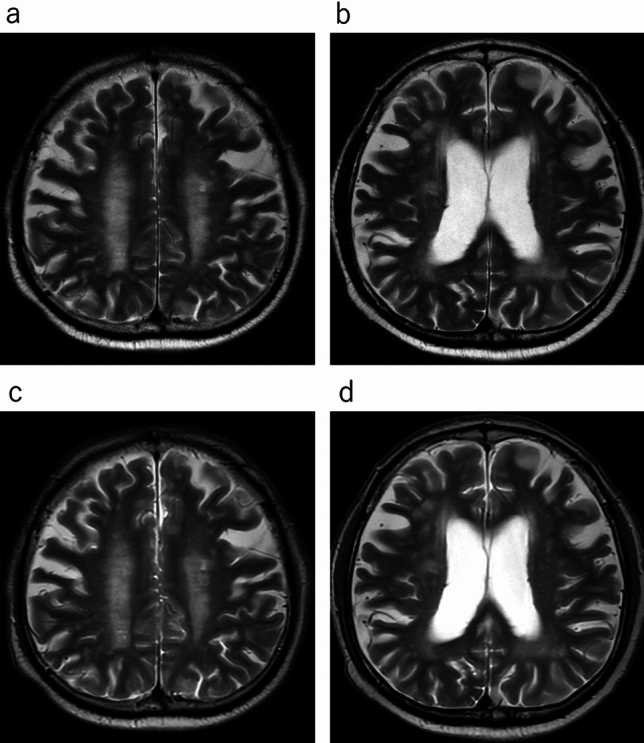
Representative case with ischemic CNS disease. Conventional T2WIs (**a, b**) and DL-based ultra-fast T2WIs (**c, d**) obtained from the same patient. The visualization of multiple lesions was found to be largely comparable between the conventional and DL-based ultra-fast T2WIs

**Fig. 3:**
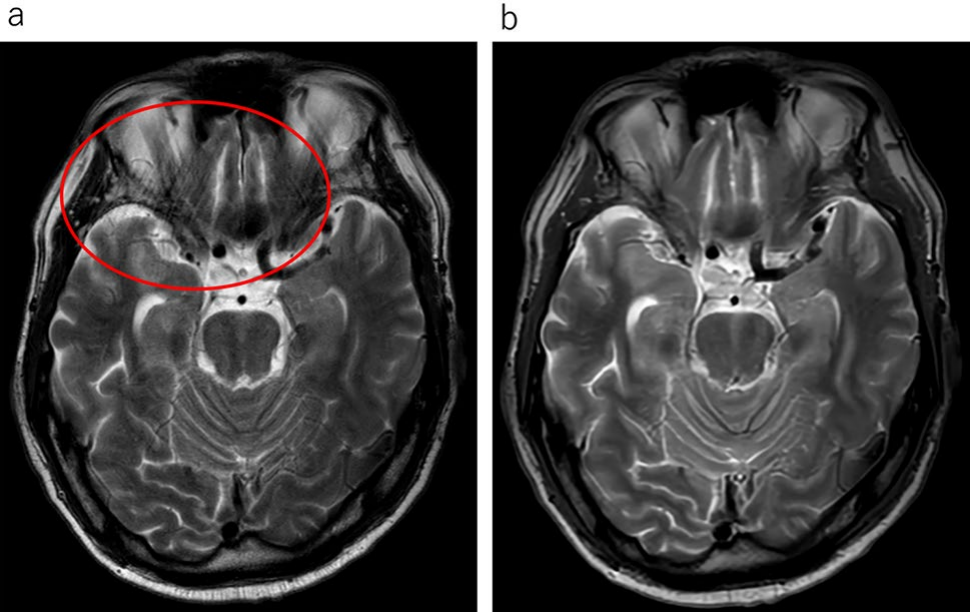
Representative case with artifact. On conventional T2WI (**a**), noise-like abnormal signals considered to be motion artifacts are observed around the skull base (*red circle*). In contrast, such artifacts were not observed on the DL-based ultra-fast T2WI (**b**). This was likely due to reduced motion during the shorter acquisition time

**Fig. 4:**
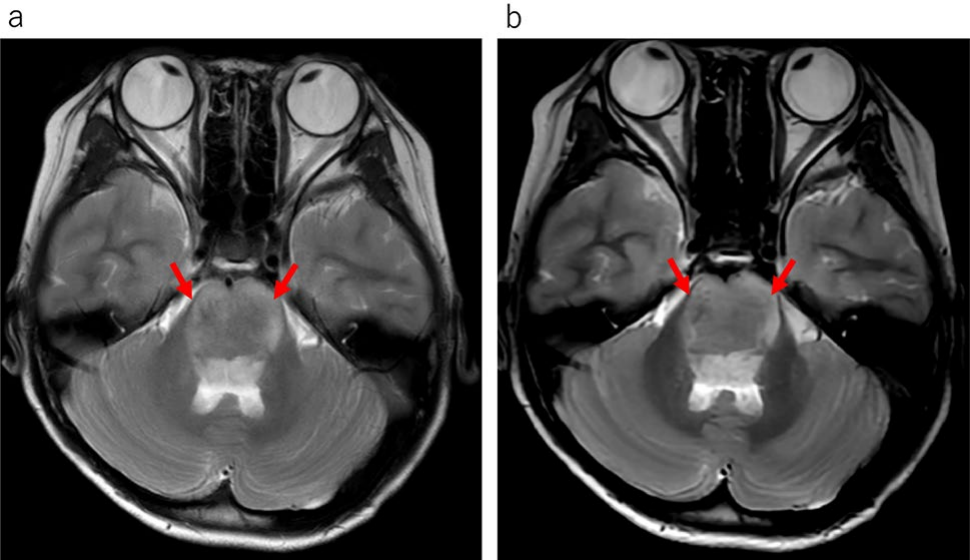
Representative case of pontine glioma. The glioma was observed as hyperintense on both conventional T2WI (**a**: *arrows*) and DL-based ultra-fast T2WI (**b**: *arrows*). The lesion depiction on DL-based ultra-fast T2WI is comparable to that on conventional T2WI, providing essentially the same diagnostic information

**Fig. 5:**
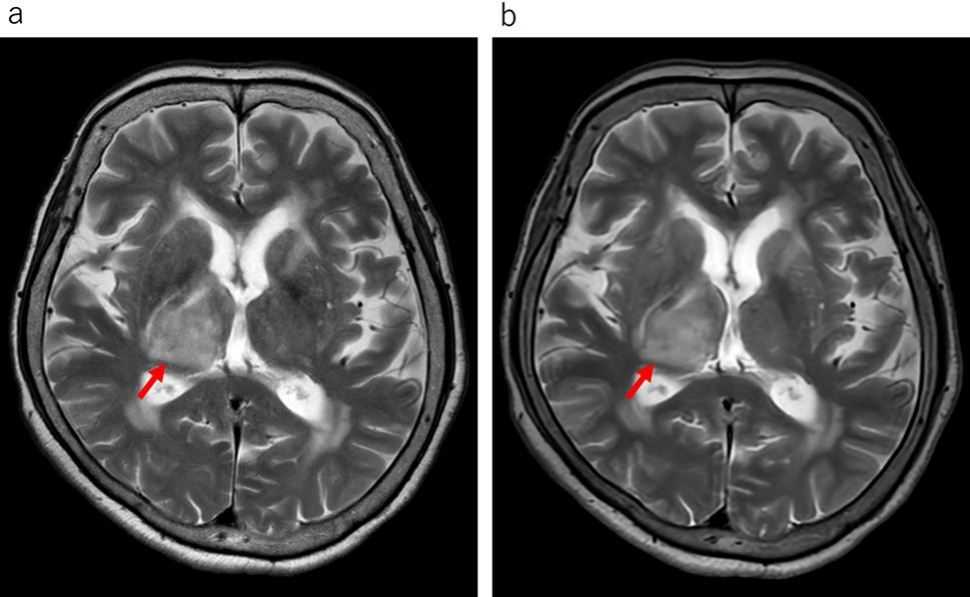
Representative case of malignant lymphoma in the right thalamus. The hyperintense lesion is depicted equivalently on both conventional T2WI (**a**: *arrow*) and DL-based ultra-fast T2WI (**b**: *arrow*)

In the qualitative analyses, significant differences were observed between the uses of conventional T2WI and UF-T2WI with DL in the overall image quality, the degree of noise, and the degree of artifacts; both Reader 1 and Reader 2 rated UF-T2WI significantly higher (all *p* < 0.001). Regarding the visibility of anatomical structures, the UF-T2WI score was significantly higher than that of the conventional T2WI in the rating by Reader 2, whereas a non-significant difference was observed by Reader 1. In cases with CNS diseases on conventional T2WI, the visibility of almost all of the lesions was evaluated as nearly identical or equivalent (i.e., 3 or 4 points) in UF-T2WI. The results of all qualitative analyses are summarized in Table 3. Figures 1, 2, 3, 4 and 5 present UF-T2WI and conventional T2WI images obtained from representative patients.

**Table 3:** The qualitative analysis results

	Reader 1			Reader 2		
	Conventional	UF-T2WI with DL	*p*-value	Conventional	UF-T2WI with DL	*p*-value
Degree of noise and artifact	3.16 ± 0.37	4	< 0.001	2.13 ± 0.34	4	< 0.001
Visibility of anatomical structures	4	4	–	2.05 ± 0.87	3.26 ± 0.69	< 0.001
Overall image quality	3.47 ± 0.56	3.95 ± 0.23	< 0.001	2.21 ± 0.41	3.45 ± 0.50	< 0.001
Ability to delineate lesions in CNS diseases		4			3.29 ± 0.62	

Data are mean ± std. dev. UF with DL: UF-T2WI with DL: ultra-fast T2WI with dual-type deep learning (DL)-based image reconstruction, CNS: central nervous system

### Quantitative analyses

In the quantitative analyses, all of the quantitative values did not follow a normal distribution. Significant differences were observed in both the SNRwm and SNRgm between conventional T2WI and UF-T2WI (both *p* < 0.001). In the CNRgm-wm assessment, significant differences were also observed between the conventional T2WI and UF-T2WI (*p* < 0.001). The results of all quantitative analyses are presented in Table 4.

**Table 4:** The quantitative analysis results

	Conventional	UF-T2WI with DL	*p*-value
SNRwm	24.6 ± 6.76	61.9 ± 27.2	< 0.001
SNRgm	25.4 ± 10.0	43.8 ± 24.9	< 0.001
CNRgm-wm	12.3 ± 4.74	36.7 ± 18.5	< 0.001

Data are mean ± std. dev. SNRwm: signal-to-noise ratio for white matter, SNRgm: signal-to-noise ratio for gray matter, CNRgm-wm: contrast-to-noise ratio between gray matter and white matter

## Discussion

Our results quantitatively and qualitatively demonstrated that UF-T2WI images acquired by SSTSE in a short scan time of 7 s with a dual-type DL-based image reconstruction technique achieved image quality that is comparable to or even better than that of conventional images. TSE-T2WI has been one of essential sequences and is frequently applied for brain evaluations in a routine clinical protocol. However, with this conventional method, the acquisition time is relatively long when sufficiently high spatial resolution is required; for example, such scanning sometimes results in patient motion and poor image quality. The SSTSE technique enables rapid and motion-related artifact-suppressed image acquisition, but its use has been limited due to the marked image blurring caused by the long echo-train, eventually resulting in insufficient image quality. Although setting a decreased echo-train would be effective to address this limitation, doing so causes a further loss of spatial resolution.

DL reconstruction was introduced to improve the SNR with effective denoising, and it has provided improved overall image quality, even in accelerated acquisitions. The DL methods have been applied to brain MRI, and their effectiveness has been demonstrated in several studies [6, 7, 15–22]. The dual-type DL reconstruction used in our present investigation included a super-resolution function as a second-stage processing step. In this investigation, the acquisition matrix of the DL-based UF-T2WI was relatively reduced compared to that of the conventional T2WI, to decrease the number of shots in the single-shot readout. However, due to the super-resolution post-processing, the spatial resolution and image blurring provided by the new method were well improved in such a low-spatial-resolution setting. In addition, the first-stage DL processing contributed to sufficient preservation of the SNR. In light of these results, we consider the comprehensive integration of single-shot acquisition and dual-type DL reconstruction highly effective and efficient.

The application of the SSTSE readout with DL reconstruction has been described [23–31]. Several of these prior investigations focused on abdominal half-Fourier single-shot turbo spin-echo (HASTE) sequences. Because a HASTE sequence is generally expected to exhibit heavily T2-weighted contrast, a large number of echo-trains is often not a serious problem. However, in our present study, we targeted brain T2WI, which required a wide range of T2 contrast including slightly high signal intensity. The heavily T2-weighted contrast provided by the traditional HASTE sequence is generally considered less suitable for evaluating the brain parenchyma, especially when detailed visualization of subtle anatomical structures is required, because slight hyperintensities in regions such as the gray matter need to be assessed. We, therefore, attempted to optimize the imaging parameters to reduce the number of shots for the avoidance of heavily T2-weighted contrast. We did so by decreasing the acquisition matrix, and we were subsequently able to obtain images with both sufficient spatial resolution and balanced T2-weighted contrast by applying super-resolution processing. Actually, almost all of the CNS diseases observed on the conventional T2WI were assessed as nearly identical or equivalent to those on UF-T2WI by the qualitative analysis. UF-T2WI successfully depicted CNS diseases that generally exhibited slightly-to-moderately high signal intensity on T2WI. The present study is the first to demonstrate the effectiveness of combining SSTSE with dual-type DL-based image reconstruction incorporating both denoising and super-resolution. The acquisition time of 7 s for UF-T2WI observed in this study has the potential to greatly shorten the lengthy scan times of MRI examinations and significantly change clinical workflows. The clinical value of a 7 s T2WI acquisition is expected to extend beyond routine brain MRI examinations to emergent cases such as acute stroke and trauma, as shorter scan times in these situations directly contribute to earlier treatment. In contrast, we need to pay careful attention to DL-related artifacts, such as pseudolesions that may result from over-denoising or over-smoothing, although no such artifacts were observed in our study. As SSTSE involves aggressive undersampling, the possibility of DL-related artifacts cannot be fully excluded and should be carefully considered in daily clinical image interpretation. Furthermore, there are also important aspects for clinical use. DL-processed images may differ somewhat in quality from conventional images, and some radiologists might still prefer the familiar appearance obtained by the conventional method. A period of training and adjustment may help radiologists become familiar with DL-processed images and support their broader clinical implementation.

Our study has several limitations. First, the number of patients was relatively small (*n* = 38) and they were drawn from a single institution. Our findings should thus be treated as preliminary. Second, the cases were selected from examinations that had been performed using a specific MRI scanner during a fixed period, which may have introduced selection bias. Third, future studies should also be conducted to evaluate the CNR and SNR of pathological lesions, to determine whether UF-T2WI can provide reliable results for quantitative assessment across various lesion types. In addition, in the present study, we evaluated only normal anatomical structures and several CNS diseases on brain T2WI using a specific 3 T MRI scanner. Future studies should investigate the utility of 1.5 T systems and, as mentioned above, include a wide variety of pathological lesions. In this regard, it may be necessary to further expand and update the training dataset of the DL model.

In conclusion, the use of a single-shot technique combined with dual-type DL reconstruction with the super-resolution processing enabled the acquisition of clinically acceptable images within an ultra-short scan time of 7 s. This method has the potential to greatly shorten MRI acquisition times and significantly improve clinical workflows.
